# Formulation of a spatiotemporal model for the analysis of neonatal mortality amidst SDG interventions: The case of Uganda

**DOI:** 10.1371/journal.pone.0323859

**Published:** 2026-03-19

**Authors:** George Bamwebaze, Gichuhi Anthony Waititu, Richard O. Awichi, Atinuke Olusola Adebanji

**Affiliations:** 1 Department of Mathematics and Data Science, Pan African University Institute for Basic Science Technology and Innovation, Nairobi, Kenya; 2 Department of Statistics and Actuarial Sciences, Jomo Kenyatta University of Agriculture and Technology, Nairobi, Kenya; 3 Department of Mathematics and Statistics, Kyambogo University, Kampala, Uganda; 4 Department of Statistics and Actuarial Science, Kwame Nkrumah University of Science and Technology, Kumasi, Ghana; King Faisal University, SAUDI ARABIA

## Abstract

This study aimed to formulate a dynamic linear model within a Bayesian framework to conduct a spatiotemporal analysis of neonatal mortality in Uganda during SDG interventions. This study formulated a model based on appropriate health-related covariates while considering the spatial and temporal dimensions of the data whose variable of interest (dependent variable) was a quantitative variable measuring the monthly rates of neonatal mortality (number of newborns dying within their first 28 days of life) at the district level. Through Markov chain Monte Carlo (MCMC) simulations, the applicability of the model could be assessed using simulated data covering 14 years, starting in January 2010, to evaluate the situation before and after the implementation of interventions to achieve the SDGs targets. Using a Bayesian approach through the Kalman filtering technique, the parameters of the formulated model were estimated. This study used the same technique through Gibbs sampling to extract meaningful information from the simulated data and provide reliable forecasts for the rates of neonatal mortality.

## Introduction

The burden of neonatal mortality has continued to increase in most countries [1]. A healthy economy with a minimal mortality rate is desirable for every country worldwide. Neonatal (newborn) mortality is defined for this study as death within the first 28 days of life per 1000 live births as established by the World Health Organization [2]. As noted by [1], reducing neonatal mortality is an essential part of SDG 3, Section 3.2.2: *countries should aim to reduce neonatal mortality to at least 12 per 1,000 live births and under five mortality to at least 25 per 1,000 live births by 2030* and achieving this requires an understanding of the levels of and trends in neonatal mortality. According to a study by [3], 5.2 million children died before reaching their fifth birthday in 2019, with almost half of those deaths, 2.4 million occurring in the first month of life, despite the countries’ efforts to reduce death rates, for instance, UN member states’ interventions in terms of the MDGs and SDGs.

In Uganda and globally, most studies on neonatal mortality, such as [4–6], have focused on risk factors while ignoring progress and forecasting the situation given that the targeted 2030 to achieve the SDGs is fast approaching. Moreover, these studies did not consider the influence of time or space. This study aimed to formulate a dynamic linear model to analyze neonatal mortality using a case study of Uganda to simultaneously investigate its persistent patterns over time and space and illuminate any unusual patterns. As mentioned by [7], the majority of time series models include well-known models such as autoregressive (AR) and moving average (MA) models. The autoregressive integrated moving average (ARIMA) and autoregressive moving average (ARMA) are helpful for handling stationary data; otherwise, they become limited. This is further supported by [8–12], as they suggest that state space models offer a very rich class of models that have several advantages. For example, they do not require stationarity, which eliminates the need to transform the data since data transformation leads to the loss of some important components in the data, which at times leads to less accurate results. To formulate a dynamic linear model, this study simulated data for health care-related factors and health care policies that were put in place as a way of achieving SDG 3.2. The health policies used are shown in Table 1.

**Table 1:** Policies in place to reduce neonatal mortality in Uganda.

Health Care Policy	Aim	Starting Year
Reproductive, Maternal, Newborn, Child, and Adolescent Health (RMNCAH) Sharpened Plan	Accelerating progress in reducing maternal and neonatal mortality by focusing on high-impact interventions and improving service delivery at all health system levels.	2016
National Health Sector Development Plan (NHSDP)	Consists of comprehensive strategies to improve health service delivery, with specific targets for reducing neonatal and maternal mortality rates through enhanced health care infrastructure, the workforce, and community health initiatives.	2015/16
Every Newborn Action Plan (ENAP)	A global initiative adopted by Uganda to end preventable neonatal deaths. It focuses on improving the quality of care during childbirth and the immediate postnatal period.	2015
Saving Mothers, Giving Life (SMGL) Initiative	The aim is to reduce maternal and neonatal mortality through improved health care delivery, infrastructure, and community engagement.	2012 (2017–Phase II)
Uganda National Newborn Steering Committee	Established to oversee and coordinate efforts to reduce neonatal mortality, ensuring alignment with national and international policies and programs.	2016
Maternal and Perinatal Death Surveillance and Response (MPDSR)	The aim is to improve the quality of maternal and newborn health services through systematic review and response to maternal and perinatal deaths, enhancing accountability and quality of care.	2015
Quality Improvement Initiative for Newborn Health (QUIN-H)	The focus is on improving the quality of neonatal care in health facilities by implementing standards and guidelines, training health care workers, and strengthening health systems.	2016

## Materials and methods

### Study design and overview

In this study, a simulation-based design was used to illustrate the application of the formulated Bayesian dynamic linear model (BDLM) in evaluating spatiotemporal trends in neonatal mortality and the impact of health policy interventions aligned with the Sustainable Development Goals (SDGs) framework. The simulated data represent monthly neonatal mortality rates and related health indicators in Uganda from January 2010 to December 2023, reflecting the influence of key health care initiatives implemented during the SDGs period. The rationale for choosing this period was to cover the time before and after the implementation of interventions to achieve the SDGs and to assess their impact.

### Methodological framework

**Fig 1:**
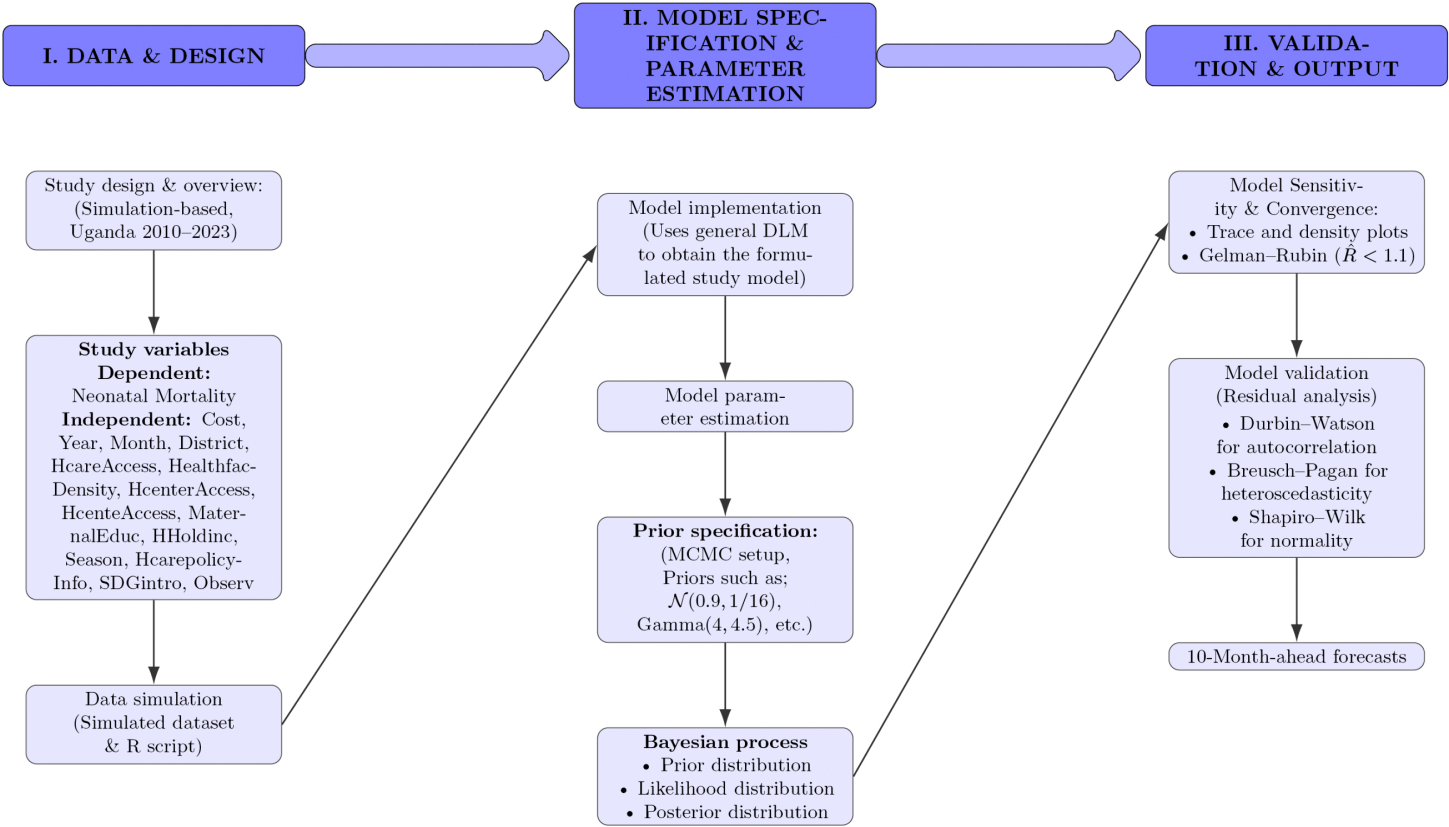
Methodological flowchart summarizing the data design, Bayesian model specification & parameter estimation, and validation process.

To make the methodology presentation easier to follow, this study constructed a visual diagram, as shown in Fig 1.

### Study variables

The dependent variable was neonatal status, which was measured as the monthly number of liveborn children who died within their first 28 days of life per district.

The independent variables (covariates) were as follows:

1. Cost was measured as the monthly amount of money the district receives from the Ministry of Finance through the Ministry of Health for the implementation of the seven policies obtained by dividing the annual amount by 12.
2. Year was measured as the calendar year for which the data were recorded.
3. Month was measured as the month of the calendar year for which the data were recorded.
4. District was measured as the name of the district from which the data were recorded.
5. Region was measured as the main administrative region in which the district was located since these data are reported monthly from districts but not regions. Therefore, districts were regrouped to obtain regions during data cleaning and editing. The regions were coded as 1 for Central, 2 for Western, 3 for Eastern and 4 for Northern.
6. HealthfacDensity was measured as the number of health centers in the district.
7. HcareAccess was measured as the number of government health care providers in the district.
8. HcenterAccess was measured as the distance in meters to the nearest health center from the farthest household in the district.
9. MaternalEduc was measured as the average number of antenatal visits by women in the district per month.
10. HholdInc was measured as the average monthly household income in the district.
11. Seasons were measured as the changes in weather conditions in the country captured as (1 Dry, 0 Wet).
12. SDGintro was measured as the time of the start of the SDGs during the observation period was captured as (1 After, 0 Before).
13. HcarepolicyInfo was measured as the time of the start of the 7 health care policies shown in Table 1 during the observation period captured as (1 After, 0 Before).
14. Observ was measured as the observation time running from 1 in January 2010 to December 2023.

### Data simulation procedures and availability

A synthetic time series was generated through simulations to emulate realistic variations in neonatal mortality associated with health care system performance and policy interventions. A simulated dataset comprising 1,008 observations, incorporating health system indicators such as antenatal care coverage, facility access, and skilled birth attendance, along with temporal components that capture secular trends and seasonal fluctuations, was used. This reflects Uganda’s neonatal mortality context before and during SDG implementation, using baseline mortality of 27 deaths per 1,000 live births based on a report by the Ministry of Health, Uganda, as reported in [15]. We also based on Sample data from the DHIS2 (District Health Information System 2) since this was reported as a better reporting tool by [17] and the UN Inter-agency Group for Child Mortality Estimation (UN IGME) [18]. The simulation was performed in R (version 4.3.2) to ensure reproducibility and control over model parameters, thereby allowing transparent evaluation of methodological performance under known data-generating conditions. The simulated dataset, along with the R script used for data generation, model estimation, validation, and figure production, is publicly accessible, as it is simulated.

#### Bayesian inference and prior specification.

Bayesian inference was performed using Markov chain Monte Carlo (MCMC) methods implemented in JAGS (Just Another Gibbs Sampler). The following prior distributions were specified:

- **Regression coefficients:**
  $$\beta_{j}\sim 𝒩(0.9,\;1/16)$$
  for the continuous variables and
  $$\beta_{j}\sim 𝒩(0.9,\;1/36)$$
  for the categorical variables.
- **Observation variance:**
$$\sigma_{\text{obs}}^{2}\sim \text{Gamma}(4.5,\;4.5)$$
- **State variance:**
$$\sigma_{\text{state}}^{2}\sim \text{LogNormal}(1,\;0.25)$$
- **Initial state:**
$$\mu_{1}\sim 𝒩(0,\;3)$$

These priors are weakly informative, chosen for their ability to identify the model without influencing posterior estimates. The scale of the priors was informed by prior neonatal mortality studies and time-series modeling literature. Four independent MCMC chains were run, each with 1,000 burn-in iterations followed by 5,000 sampling iterations. Convergence was assessed through trace plots, posterior density plots, and the Gelman–Rubin diagnostic, with $\hat{R}<1.1$ indicating satisfactory convergence. Posterior means and 95% credible intervals (CrIs) were reported for all the model parameters.

### Model implementation

#### Nature of and rationale for the model.

The study opted to formulate a dynamic linear model to monitor the impact of the SDG interventions on neonatal mortality, as dynamic linear models constitute a flexible class of models that can effectively address typical features in policy monitoring and large datasets.

For instance, this study aimed to construct a dynamic linear model to assess the situation after the onset of the SDGs, having previously evaluated the situation. To achieve this objective, this study used a general Gaussian dynamic linear model, as presented in Eq 1:


$$Y_{it}=F_{xit}Q_{it}+v_{t}$$ (1)

$$Q_{it}=G_{t}Q_{it-1}+\omega_{t}$$

where $Y_{it}$ represents the observation for the $i^{th}$ state at time t.

$F_{xit}$ denotes a function of the *x* covariates for the $i^{th}$ state at time t specified as


$$f(x_{11},x_{12},\ldots ,x_{it})=\sum_{i=1}^{n}\sum_{t=1}^{h}x_{it}$$ (2)

$Q_{it}$ is a vector of time-carrying parameters

$G_{t}$ is a transition matrix depicting how the model parameters evolve over time.

$v_{t}\sim 𝒩(0,V_{i,t})$ and $\omega_{t}\sim 𝒩(0,W_{i,t})$ are the observation and evolution errors, respectively.

On the basis of (1), the model is formulated as follows (3):


$$Y_{it}=\beta_{0t}+\sum_{i=1}^{n}\sum_{m=1}^{nm}\sum_{t=1}^{h}\beta_{imt}x_{imt}+\sum_{i=1}^{n}\sum_{m=1}^{nm}\sum_{t=1}^{h}\beta_{im(m+1)t}x_{imt}x_{im(m+1)t}+\epsilon_{t}$$ (3)

$$[\begin{array}{c} \beta_{0t}\\ \beta_{11t}\\ \beta_{112t}\\ \vdots \\ \beta_{nmh}\\ \beta_{nm(m+1)h} \end{array}]=[\begin{array}{c} \beta_{0(t-1)}\\ \beta_{1m(t-1)}\\ \beta_{112(t-1)}\\ \vdots \\ \beta_{nm(h-1)}\\ \beta_{nm(m+1)(h-1)} \end{array}]+\omega_{t}$$

Where:

$Y_{it}$ represents the $i^{th}$ district neonatal mortality rate at time t.

$x_{11},x_{12},\ldots ,x_{nh}$ are the m covariates, including the maternal health care policies and interventions, in which case a code 0 is used for Before and 1 for After the introduction of a given policy/intervention during the observation time for a given policy or intervention.

$x_{imt}x_{im(m+1)t}$ is the interaction covariate between the $m^{th}\;and\;(m+1{)}^{th}$ covariate, including the maternal health care policies and interventions, with $\beta_{im(m-1)t}$ as their respective coefficients,

$\beta_{0t}$ denotes the time-varying intercept.

$\beta_{111},\beta_{121},\ldots ,\beta_{nmh}$ are the time-varying coefficients associated with each of the m covariates, including maternal health care policies and interventions.

$\epsilon_{t}$ and $\omega_{t}$ are the observation and evolution error terms, respectively, where $\epsilon_{t}\sim 𝒩(0,V_{t})$ and $\omega_{t}\sim 𝒩(0,W_{t})$.

#### Model parameter estimation.

A Bayesian approach using the Kalman filtering technique was used for estimating the study model parameters because the Bayesian approach focuses on how a particular part of the data depends on the other data parts since this study had to compare the neonatal mortality situation in the country before and after the introduction of the SDGs, implying that past values had a considerable influence on future values in this study.

Considering that Bayesian inference has its roots in Bayes rule, as noted in the studies by [13] and [14], to obtain the final parameter estimates, this study made use of the theorem through the posterior density function as expressed in (4);


P(β|Y)∝P(Y|β)P(β) (4)

The final parameter estimates are obtained using a quadratic mean loss function (squared error loss) method, which involves obtaining a full posterior distribution of parameters and then calculating their average as the final parameter estimate.

Rewriting the formulated model by the study presented in (3), which is expressed in the form of two equations, observation and state equations, we obtain (5);


$$Y_{t}=X_{t}^{T}\beta_{t}+\epsilon_{t}$$ (5)

$$\beta_{t}=\beta_{t-1}+\omega_{t}$$

where

$Y_{t}$ is the observed data ${\text{X}}_{t}$ is a p-dimensional design vector of covariates and the interaction terms.

$\beta_{t}$ is a p-dimensional time-varying parameter vector (for both covariates and the interaction terms).

$\epsilon_{t}$ and $\omega_{t}$ are the observation and evolution error terms, respectively, where $\epsilon_{t}\sim 𝒩(0,V_{t})$ and $\omega_{t}\sim 𝒩(0,W_{t})$.

Since the formulated model is a multivariate Gaussian state space model, based on the standard results of a multivariate Gaussian distribution pertaining to its marginal and conditional distributions, the random vector of parameters $\beta_{t}$ also has a Gaussian distribution, and the same applies to the other respective marginal and conditional distributions.

Therefore, since all the relevant distributions are Gaussian, and bearing in mind that the process of Bayesian inference involves passing from a prior distribution, P(β), to a posterior distribution, P(β|Y), we estimated the model parameters through the Kalman filtering technique to obtain the means and variances to finally derive the parameter estimates (posterior means) through the following procedure:

- Obtaining the prior distribution (based on the evolution equation reflected in the second line of (3)).

Considering the fact that a dynamic linear model is a Gaussian time space model, we used a normal prior distribution for a p-dimensional state vector by first obtaining the initial values where $\hat{\beta_{0}}\sim 𝒩(m_{0},c_{0})$, and we then attained one step ahead of the prediction of the parameter vector distribution ${\hat{\beta }}_{t}|y_{1:t-1}$ by first obtaining the mean as given in (6)


$$\begin{array}{c} \begin{array}{cc} {\hat{\beta }}_{t|t-1} & =E(\beta_{t}|y_{1:t-1})\\ & =E(E(\beta_{t}|\beta_{t-1},y_{1:t-1})|y_{1:t-1})\\ & =E(\beta_{t-1}|y_{1:t})\\ & =m_{t-1} \end{array} \end{array}$$ (6)

Then, we obtained the predicted covariance in (7) denoted as $c_{t|t-1}$


$$\begin{array}{c} \begin{array}{cc} c_{t|t-1} & =Var(\beta_{t}|y_{1:t-1})\\ & =E(Var(\beta_{t}|\beta_{t-1},y_{1:t-1})|y_{1:t-1})+Var(E(\beta_{t}|\beta_{t-1},y_{1:t-1})|y_{1:t-1})\\ & =W_{t}+c_{t-1} \end{array} \end{array}$$ (7)

Therefore, ${\hat{\beta }}_{t}\sim 𝒩(m_{t-1},(W_{t}+c_{t-1}))$ which is the prior distribution.

Since our prior has been derived on the basis of a normal distribution, we can write its probability density function as given in (8):


$$P(\beta_{t})=\frac{1}{\sqrt{2\pi (W_{t}+c_{t-1}{)}^{2}}}\exp-\frac{(\beta_{t}-m_{t-1}{)}^{2}}{2(W_{t}+c_{t-1}{)}^{2}}$$ (8)

ii Obtaining the likelihood distribution function (based on the observation equation reflected in the first line of (3)).

We start this step by obtaining the predicted (one step) distribution function of $Y_{t}$ (the observed value): $Yt|y_{1:t-1}$

Let $Yt|y_{1:t-1}\sim 𝒩(n_{t},s_{t})$

Nevertheless, we first obtained the mean, $n_{t}$


$$\begin{array}{c} \begin{array}{cc} n_{t} & =E(Y_{t}|y_{1:t-1})\\ & =E(E(Y_{t}|\beta_{t},y_{1:t-1})|y_{1:t-1})\\ & =E((X_{t}^{T}\beta_{t})|y_{1:t-1})\\ & =X_{t}^{\prime }\beta_{t} \end{array} \end{array}$$ (9)

We then obtained the variance, $s_{t}$


$$\begin{array}{c} \begin{array}{cc} s_{t} & =Var(Y_{t}|y_{1:t-1})\\ & =E(Var(Y_{t}|\beta_{t},y_{1:t-1})|y_{1:t-1})+Var(E(Y_{t}|\beta_{t},y_{1:t-1})|y_{1:t-1})\\ & =V_{t}+X_{t}c_{t|t-1}X_{t}^{\prime } \end{array} \end{array}$$ (10)

Therefore, $Y_{t}\sim 𝒩(X_{t}^{\prime }\beta_{t},(V_{t}+X_{t}c_{t|t-1}X_{t}^{\prime }))$, which is the observed value likelihood distribution.

Since the observed value variable follows a normal distribution, we can write its likelihood function probability density function as follows;


$$P(Y_{t}|\beta_{t})=\frac{1}{\sqrt{2\pi (V_{t}+X_{t}c_{t|t-1}X_{t}^{\prime }{)}^{2}}}\exp-\frac{(Y_{t}-X_{t}^{\prime }\beta_{t}{)}^{2}}{2(V_{t}+X_{t}c_{t|t-1}X_{t}^{\prime }{)}^{2}}$$ (11)

iii Obtaining the posterior distribution

From (4), an expression derived from the Bayes theorem, we can obtain the posterior probability density function by multiplying (11) by (8) and hence obtaining the probability density function of the posterior distribution function as given in (12);


$$P(\beta_{t}|Y_{t})=\frac{1}{\sqrt{2\pi (W_{t}+c_{t-1}{)}^{2}(V_{t}+X_{t}c_{t|t-1}X_{t}^{\prime }{)}^{2}}}\exp\left\{-\frac{(\beta_{t}-m_{t-1}{)}^{2}}{2(W_{t}+c_{t-1}{)}^{2}}-\frac{(Y_{t}-X_{t}^{\prime }\beta_{t}{)}^{2}}{2(V_{t}+X_{t}c_{t|t-1}X_{t}^{\prime }{)}^{2}}\right\}$$ (12)

Since both the prior and likelihood distributions are Gaussian, the resulting posterior is also Gaussian. If we let the posterior $\beta_{t}|Y_{t}\sim 𝒩(d_{t},p_{t})$, from the first property of a Gaussian distribution pertaining to the probability density function, we can obtain the values of $d_{t}\;,\;p_{t}$ by simplifying the right-hand side of (12);


$$P(\beta_{t}|Y_{t})=\frac{1}{\sqrt{2\pi \;(W_{t}+c_{t-1}{)}^{2}\;(V_{t}+X_{t}c_{t|t-1}X_{t}^{'}{)}^{2}}}\;\;\;\;\times \exp\;\left[-\frac{(\beta_{t}-m_{t-1}{)}^{2}}{2\;(W_{t}+c_{t-1}{)}^{2}}-\frac{(Y_{t}-X_{t}^{'}\beta_{t}{)}^{2}}{2\;(V_{t}+X_{t}c_{t|t-1}X_{t}^{'}{)}^{2}}\right]\;=\frac{1}{\sqrt{2\pi \;(W_{t}+c_{t-1}{)}^{2}\;(V_{t}+X_{t}c_{t|t-1}X_{t}^{'}{)}^{2}}}\exp\;\left(-\frac{(Y_{t}-X_{t}^{'}\beta_{t}{)}^{2}}{2\;(V_{t}+X_{t}c_{t|t-1}X_{t}^{'}{)}^{2}}\right)$$ (13)

From (13), since the posterior $\beta_{t}|Y_{t}\sim 𝒩(d_{t},p_{t})$, it implies that;


$$d_{t}=\epsilon_{t},\;p_{t}=(W_{t}+c_{t-1}{)}^{2}(V_{t}+X_{t}c_{t|t-1}X_{t}^{\prime }{)}^{2}=(V_{t}+X_{t}c_{t|t-1}X_{t}^{\prime }{)}^{2}=(W_{t}+c_{t-1}{)}^{2}$$

Therefore, $\beta_{t}|Y_{t}\sim 𝒩(\epsilon_{t},(W_{t}+c_{t-1}{)}^{2})$

From this expression, we used a quadratic mean loss function (squared error loss), which involves computing the full posterior distribution of the parameters and averaging them to obtain the final parameter estimate.

#### Model validation.

To validate the model, we conducted residual tests on autocorrelation, heteroscedasticity, and normality using the Durbin–Watson test, the Breusch Pagan test, and the Shapiro–Wilk test, respectively. The following properties were checked for:

Autocorrelation: The residuals should not be autocorrelated.
Homoscedasciticity: The residuals should have a constant variance.
Normality: The residuals are expected to be normally distributed.

To verify the above model properties, tests were carried out using the following hypotheses:

$H_{01}$: Residuals are not autocorrelated vs. $H_{11}$: Residuals are autocorrelated.
$H_{02}$: Residuals have constant variance Vs $H_{12}$: Residuals do not have constant variance.
$H_{03}$: Residuals are normally distributed $H_{13}$ vs. $H_{01}$: Residuals are not normally distributed.

#### Test statistics, critical values, and the decision rule.

While testing for autocorrelation of residuals, the Durbin–Watson test was used. To obtain its test statistic, the following formula was used:


$$DW=\frac{\sum_{t=2}^{T}(e_{t}-e_{t-1}{)}^{2}}{\sum_{t=1}^{T}e_{t}^{2}}$$ (14)

where

$e_{t}$ = the residuals from the regression model.
T = the number of observations.

With respect to the critical value and the decision rule, we assume that the DW (value) ranges between 0 and 4; since a DW value approaching 2 indicates no autocorrelation, a value approaching 0 indicates positive autocorrelation, and a value approaching 4 indicates negative autocorrelation, taking into consideration the probability value of the test statistic in comparison to the level of significance.

The Breusch Pagan test was used to assess the homoscedasticity of residuals, whereby to obtain its test statistic, the following formula was used:


$$BP=\frac{nR_{adj}^{2}}{2}$$ (15)

where

n = number of observations

$R_{adj}^{2}$ = R-squared value obtained by regressing the squared residuals from the original regression on the independent variables, their squares, and their cross products. This regression is known as auxiliary regression.

With respect to the critical value and the decision rule, because the test statistic of Breusch Pagan follows a chi-square distribution with degrees of freedom equal to the number of independent variables in the auxiliary regression, the test statistic value was compared with the critical (tabulated) value to ascertain whether to reject the null hypothesis basically considering the significance level and the reported probability value of the test statistic.

The Shapiro–Wilk test was used to determine if the residuals were normally distributed, whereby the following formula was used to obtain its test statistic, denoted as W:


$$W=\frac{(\sum_{i=1}^{n}a_{i}e_{\left(i\right)}{)}^{2}}{\sum_{i=1}^{n}(e_{i}-\bar{e}{)}^{2}}$$ (16)

where

$e_{\left(i\right)}$ = ordered residuals from smallest to largest.

$\bar{e}$ = mean of the residuals.

$a_{i}$ = constants derived from the expected values of the order statistics of a standard normal distribution computed using statistical software.

n = number of observations.

The critical value and the decision rule were determined on the basis that W ranges between 0 and 1; values of W close to 1 suggest that the residuals are approximately normally distributed, whereas lower values indicate deviations from normality.

Furthermore, The performance of the Bayesian dynamic linear model formulated in this study has also been evaluated in our separate, independently published study, which compares it with mixed-effects interrupted time-series models using real-world neonatal mortality data from Uganda (2015–2023). Whereas the present work emphasizes model formulation and Monte Carlo simulation-based validation, the other study focuses on empirical comparison [16].

### Model sensitivity analysis

To assess model fitness, a residual analysis was performed. Having estimated posterior means and credible intervals for the model parameters, trace and means plots were constructed, which enabled convergence to be assessed. To evaluate predictive performance, the model was extended to generate forecasts of neonatal mortality for the first 10 months after December 2023, aligned with the national health planning cycles. In addition to using trace plots as a way of assessing model convergence visually and numerically, convergence was assessed using the Gelman–Rubin diagnostic ($\hat{R}$), with values less than 1.1 indicating satisfactory convergence.

## Results and discussion

### Model validation

The following conclusions have been made regarding model validation on the basis of the results in Table 2:

**Table 2:** Models validation tests results.

Model Test	Test Statistic	p-value
Durbin Wartson Test for Autocorrelation	0.0725	0.0000
Studentized Breusch–Pagan Test for Heteroscedasticity	11.8440	0.3755
Shapiro–Wilk normality test	0.9963	0.0164

Results of diagnostic tests assessing model assumptions. The tests include checks for autocorrelation (Durbin–Watson), homoscedasticity (Breusch–Pagan), and normality (Shapiro–Wilk).

While testing for autocorrelation of residuals with the Durbin-Watson test, though the Durbin-Watson value (DW = 0.07253) ranges between 0 and 2, showing no autocorrelation of the residuals, its p-value ≤ 0.05, indicating rejection of the null hypothesis, which implies residuals are autocorrelated.

While testing for homoscedasticity with the Breusch-Pagan test, because the p-value (0.3755) is greater than 0.05, we fail to reject the null and conclude that the residuals have constant variance.

While testing whether the residuals are normally distributed with the Shapiro–Wilk test, based on the fact that Shapiro–Wilk’s test value W ranges between 0 and 1, since W (W = 0.9963) is close to or equal to 1, this suggests that the residuals are normally distributed but its p-value is less than 0.05 which implies rejection of the null hypothesis and hence concluding that residuals aren’t normally distributed.

The model fails to fulfill the assumptions of non-autocorrelation and normality of residuals based on the probability values, but this does not invalidate our model, but instead gives justification for its use based on the fact that unlike classical linear regression, Bayesian inference does not rely on independence or normality assumptions for valid parameter estimation. Instead, such diagnostics highlight temporal dependence and unobserved heterogeneity in the data, which motivate the use of a Bayesian framework capable of explicitly modeling complex data-generating processes and propagating uncertainty through the posterior distribution [19–22]

Findings from [16] show that the Bayesian Dynamic Linear Model outshone Mixed Effects Interrupted Time Series Model as a preferred model for Neonatal mortality analysis amidst SDGs onset impact evolution

### Model convergence

**Fig 2:**
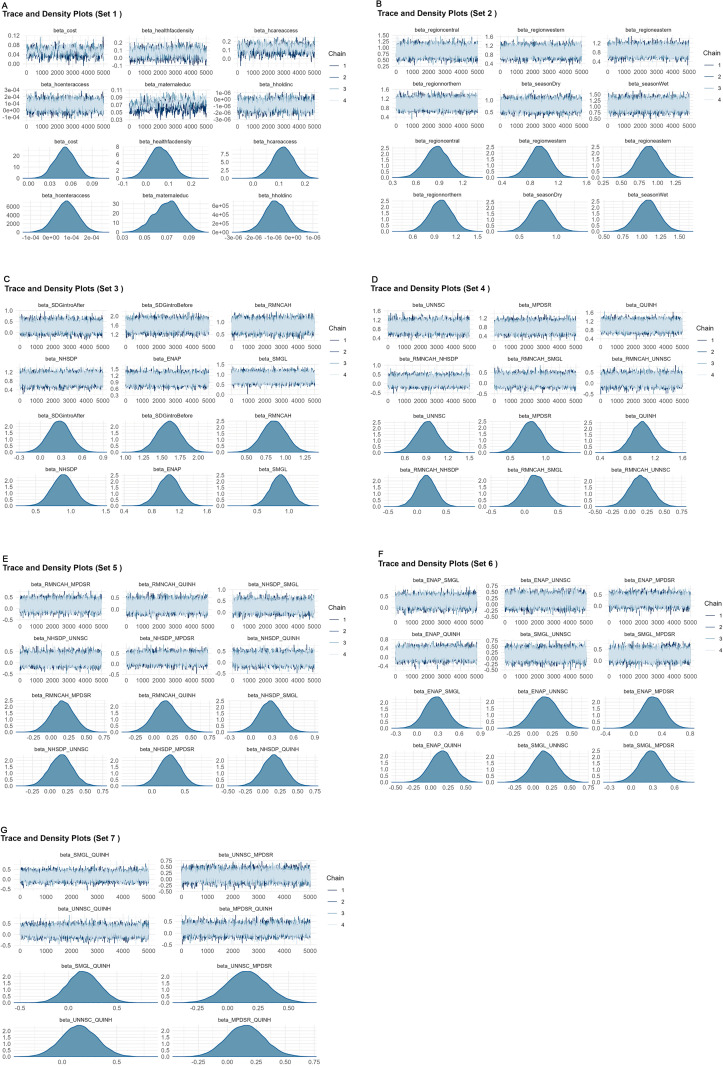
Model diagnostics using trace and density plots for posterior distributions showing MCMC convergence of model parameters: (in Fig 2(2A)–2(2G) ((2A)) Trace and density plots for beta_cost, beta_healthfacdensity, beta_hcareaccess, beta_hcenteraccess, beta_maternaleduc and beta_hholdinc. (Set 1). ((2B)) Trace and density plots for beta_regioncentral, beta_regionwestern, beta_regioneastern, beta_regionnorthern, beta_seasonDry and beta_seasonWet. (Set 2). ((2C)) Trace and density plots for beta_SDGintroAfter, beta_SDGintroBefore, beta_RMNCAH, beta_NHSDP, beta_ENAP, and beta_SMGL. (Set 3). ((2D)) Trace and density plots for beta_UNNSC, beta_MPDSR, beta_QUINH, beta_RMNCAH_NHSDP, beta_RMNCAH_SMGL and beta_RMNCAH_UNNSC. (Set 4). ((2E)) Trace and density plots for beta_RMNCAH_MPDSR, beta_RMNCAH_QUINH, beta_NHSDP_SMGL, beta_NHSDP_UNNSC, beta_NHSDP_MPDSR and beta_NHSDP_QUINH. (Set 5). ((2F)) Trace and density plots for beta_ENAP_SMGL, beta_ENAP_UNNSC, beta_ENAP_MPDSR, beta_ENAP_QUINH, beta_SMGL_UNNSC and beta_SMGL_MPDSR. (Set 6). ((2G)) Trace and density plots for beta_SMGL_QUINH, beta_UNNSC_MPDSR, beta_UNNSC_QUINH and beta_MPDSR_QUINH. (Set 7).

As shown in Fig 2 depicted in Fig 2(2A)–2(2G), the trace and density plots indicate convergence across almost all parameters except one MaternalEduc, measuring the number of women doing their first antenatal visit per month in the district, whose Gelman–Rubin diagnostic ($\hat{R}$) statistic (1.37) is greater than the threshold value of 1.1, as further reported by the Gelman–Rubin diagnostic ($\hat{R}$) results in Table 3. This implies that the MCMC algorithm successfully captured the true posterior distribution of our model parameters, meaning that our parameter estimates, uncertainties, and forecasts are trustworthy. We have also included in the Supporting Information a file containing the graphical illustration of the convergence test obtained from Table 3.

**Table 3:** Gelman–Rubin diagnostic ($\hat{R}$) and 95% upper credible intervals for model parameters.

Parameter	Rhat	Upper CI
beta_ENAP	1.000175	1.000347
beta_ENAP_MPDSR	1.000463	1.001596
beta_ENAP_QUINH	1.000501	1.001577
beta_ENAP_SMGL	1.000462	1.001638
beta_ENAP_UNNSC	1.000261	1.001014
beta_MPDSR	1.000747	1.002507
beta_MPDSR_QUINH	1.000233	1.000856
beta_NHSDP	1.000417	1.001231
beta_NHSDP_MPDSR	1.000320	1.001285
beta_NHSDP_QUINH	1.000167	1.000564
beta_NHSDP_SMGL	0.999963	1.000094
beta_NHSDP_UNNSC	1.000676	1.002274
beta_QUINH	0.999980	1.000235
beta_RMNCAH	1.000159	1.000641
beta_RMNCAH_MPDSR	1.000880	1.002851
beta_RMNCAH_NHSDP	1.000047	1.000434
beta_RMNCAH_QUINH	1.000010	1.000222
beta_RMNCAH_SMGL	1.000066	1.000274
beta_RMNCAH_UNNSC	0.999997	1.000199
beta_SDGintroAfter	1.000290	1.000782
beta_SDGintroBefore	1.000657	1.002164
beta_SMGL	1.000029	1.000381
beta_SMGL_MPDSR	1.000483	1.001701
beta_SMGL_QUINH	1.000113	1.000638
beta_SMGL_UNNSC	1.000513	1.001764
beta_UNNSC	1.000309	1.000839
beta_UNNSC_MPDSR	1.000839	1.002859
beta_UNNSC_QUINH	1.000633	1.002171
beta_cost	1.034672	1.100494
beta_hcareaccess	1.028090	1.080495
beta_hcenteraccess	1.002710	1.008609
beta_healthfacdensity	1.027684	1.081257
beta_hholdinc	1.003082	1.009698
beta_maternaleduc	1.372455	1.884943
beta_regioncentral	1.000311	1.001073
beta_regioneastern	1.000205	1.000758
beta_regionnorthern	0.999964	1.000170
beta_regionwestern	1.001947	1.006282
beta_seasonDry	1.001102	1.003636
beta_seasonWet	1.001518	1.004688

Values of the Gelman–Rubin diagnostic ($\hat{R}$) below 1.1 indicate satisfactory convergence of the Markov chain Monte Carlo (MCMC) simulations across all model parameters. The 95% upper credible intervals provide an additional measure of convergence stability for each estimated parameter in the Bayesian dynamic linear model.

### Parameter estimates

From the means (parameter estimates) and quantiles of the parameters reported in Tables 4 and 5, most of the variables have a positive influence on the dependent variable (neonatal mortality) with the exemption of health center access and household income as reported under means whereas regarding the quantiles, most of the variables significantly influence the outcome variables since their extreme quantiles are nonzero. On the basis of the simulated data, none of the health care policies were found to interact with one another in terms of the influence of neonatal mortality.

**Table 4:** Empirical mean and standard deviation for each variable plus the standard error of the mean for model parameters estimated using the Bayesian dynamic linear model (BDLM).

Variable	Parameter Estimate	Stadard Deviation	Stadard Error
ENAP	1.1520	0.0018	0.00224
ENAP MPDSR	0.2331	0.3013	0.0053
ENAP QUINH	0.0467	0.3097	0.0051
ENAP SMGL	0.2378	0.3051	0.0049
ENAP UNNSC	0.0498	0.3100	0.0048
MPDSR	0.0478	0.2691	0.0022
MPDSR QUINH	0.5254	0.3091	0.0049
NHSDP	0.8749	0.2718	0.0021
NHSDP MPDSR	0.2367	0.3003	0.0049
NHSDP QUINH	0.0432	0.3024	0.0049
NHSDP SMGL	0.2386	0.3031	0.0053
NHSDP UNNSC	0.0045	0.3085	0.0051
QUINH	0.9607	0.2504	0.0022
RMNCAH	0.7537	0.2552	0.0024
RMNCAH MPDSR	0.0471	0.3057	0.0045
RMNCAH NHSDP	0.0544	0.3065	0.0051
RMNCAH QUINH	0.0494	0.3081	0.0049
RMNCAH SMGL	0.0511	0.3067	0.0052
RMNCAH UNNSC	0.0474	0.3066	0.0052
SDGintroAfter	0.1773	0.3186	0.0057
SDGintroBefore	1.718	0.316	0.0063
SMGL	0.7162	0.2613	0.0021
SMGL MPDSR	0.2300	0.2995	0.0052
SMGLQUINH	0.0423	0.3133	0.0054
SMGL UNNSC	0.0465	0.3132	0.0053
UNNSC	0.8606	0.2748	0.0022
UNNSC MPDSR	0.0539	0.3134	0.0051
UNNSC QUINH	0.0464	0.3077	0.0049
cost	0.0525	0.0145	0.0007
hcareaccess	0.1181	0.0376	0.0014
hcenteraccess	0.00006	0.00005	0.000001
healthfacdensity	0.0392	0.0485	0.0016
hholdinc	−0.0000007	0.0000005	0.00000001
maternaleduc	0.0726	0.0106	0.0007
regioncentral	0.7739	0.2551	0.0027
regioneastern	0.7739	0.2583	0.0026
regionnorthern	1.084	0.2580	0.0027
regionwestern	0.8729	0.0026	0.0027
seasonDry	0.6891	0.2681	0.0041
seasonWet	1.207	0.2673	0.0041

Posterior summaries representing the empirical mean, standard deviation, and standard error of the mean for parameters estimated from the Bayesian dynamic linear model fitted to simulated neonatal mortality data (January 2010–December 2023), where the mean is the parameter estimate.

**Table 5:** Quantiles for each variable.

Variable	2.5%	50%	97.5%
ENAP	0.6432	1.1530	1.6620
ENAP MPDSR	−0.3616	0.2368	0.8220
ENAP QUINH	−0.5666	0.0495	0.6510
ENAP SMGL	−0.3624	0.2387	0.8257
ENAP UNNSC	−0.5569	0.0463	0.6692
MPDSR	−0.0146	0.5269	1.0500
MPDSR QUINH	−0.5538	0.0452	0.6590
NHSDP	0.3432	0.8751	1.4090
NHSDP MPDSR	−0.3530	0.2379	0.8216
NHSDP QUINH	−0.5551	0.0425	0.6301
NHSDP SMGL	−0.3602	0.2377	0.8344
NHSDP UNNSC	−0.5583	0.0431	0.6500
QUINH	0.4741	0.9607	1.4540
RMNCAH	0.2486	0.7533	1.2580
RMNCAH MPDSR	−0.5505	0.0466	0.6456
RMNCAH NHSDP	−0.5344	0.0542	0.6624
RMNCAH QUINH	−0.5524	0.0457	0.6589
RMNCAH SMGL	−0.5579	0.0548	0.6465
RMNCAH UNNSC	−0.5578	0.0491	0.6521
SDGintroAfter	−0.4448	0.1739	0.8022
SDGintroBefore	1.0870	1.7180	2.3230
SMGL	0.2058	0.7159	1.2330
SMGL MPDSR	−0.3487	0.2317	0.8173
SMGLQUINH	−0.5720	0.0430	0.6604
SMGL UNNSC	−0.5618	0.0464	0.6677
UNNSC	0.3275	0.8605	1.4090
UNNSC MPDSR	−0.5629	0.0565	0.6563
UNNSC QUINH	−0.5535	0.0460	0.6474
cost	2.3820	0.0525	0.0808
hcareaccess	0.0459	0.1178	0.1920
hcenteraccess	−0.000045	0.000064	0.00017
healthfacdensity	−0.0560	0.0393	0.1351
hholdinc	−0.0000017	−0.0000007	0.00000035
maternaleduc	0.0513	0.0728	0.0934
regioncentral	0.2744	0.7724	1.2720
regioneastern	0.2800	0.7822	1.2880
regionnorthern	0.5750	1.0870	1.5840
regionwestern	0.3676	0.8713	1.3830
seasonDry	0.1602	0.6897	1.2060
seasonWet	0.6842	1.2060	1.7320

Posterior quartiles representing the 2.5th, 50th, and 97.5th percentiles for the parameters estimated from the Bayesian dynamic linear model, reflecting the uncertainty in the BDLM projections.

### Forecasts

Using the formulated study model, we were able to make a 10-month prediction of Uganda’s neonatal mortality rates, as shown in Table 6. The study predicted that the neonatal mortality rate of Uganda would stand at 7.7 deaths on average in the first 10 months after December 2023.

**Table 6:** Monthly forecasts of neonatal mortality for Uganda for January–October 2024 using the formulated Bayesian dynamic linear model.

Month	Forecast
January	7.665
February	7.661
March	7.665
April	7.666
May	7.667
June	7.668
July	7.666
August	7.664
September	7.661
October	7.660

Forecasted neonatal mortality rates for Uganda for the first 10 months of 2024, based on estimates from the Bayesian dynamic linear model.

## Conclusion

A dynamic linear model that can be used to monitor health care policies individually and assess the possibility of interactions between any two policies such that the wastage of resources on duplicated policies is avoided was developed in this study. It can be used to generally assess the health care situation in a country.

Using the formulated model, the study was able to make a 10-month prediction of neonatal mortality, hence demonstrating the applicability of the model.

## Supporting information

S1 File R code script. The R script used for data processing and analysis is provided as a supplementary file named the S1 File.R. (R)

S2 File Simulated dataset. An Ms. Excel-simulated dataset used for analysis is provided as a supplementary file named S2 File.xlsx. (XLSX)

S6 FileGelman’s Rubin’s diagnosis. This is the Rubin diagnosis result. This file contains graphical plots representing the Gelman–Rubin diagnostic ($\hat{R}$) and 95% upper credible intervals for the model parameters.(PDF)
