# Supplementary material for: Formulation of a spatiotemporal model for the analysis of neonatal mortality amidst SDG interventions: The case of Uganda
Source: PLoS One. 2026 Mar 19;21(3):e0323859. doi: 10.1371/journal.pone.0323859 (PMC13001985; doi:10.1371/journal.pone.0323859)
Supplement: S6 File — (PDF) [file pone.0323859.s003.pdf]

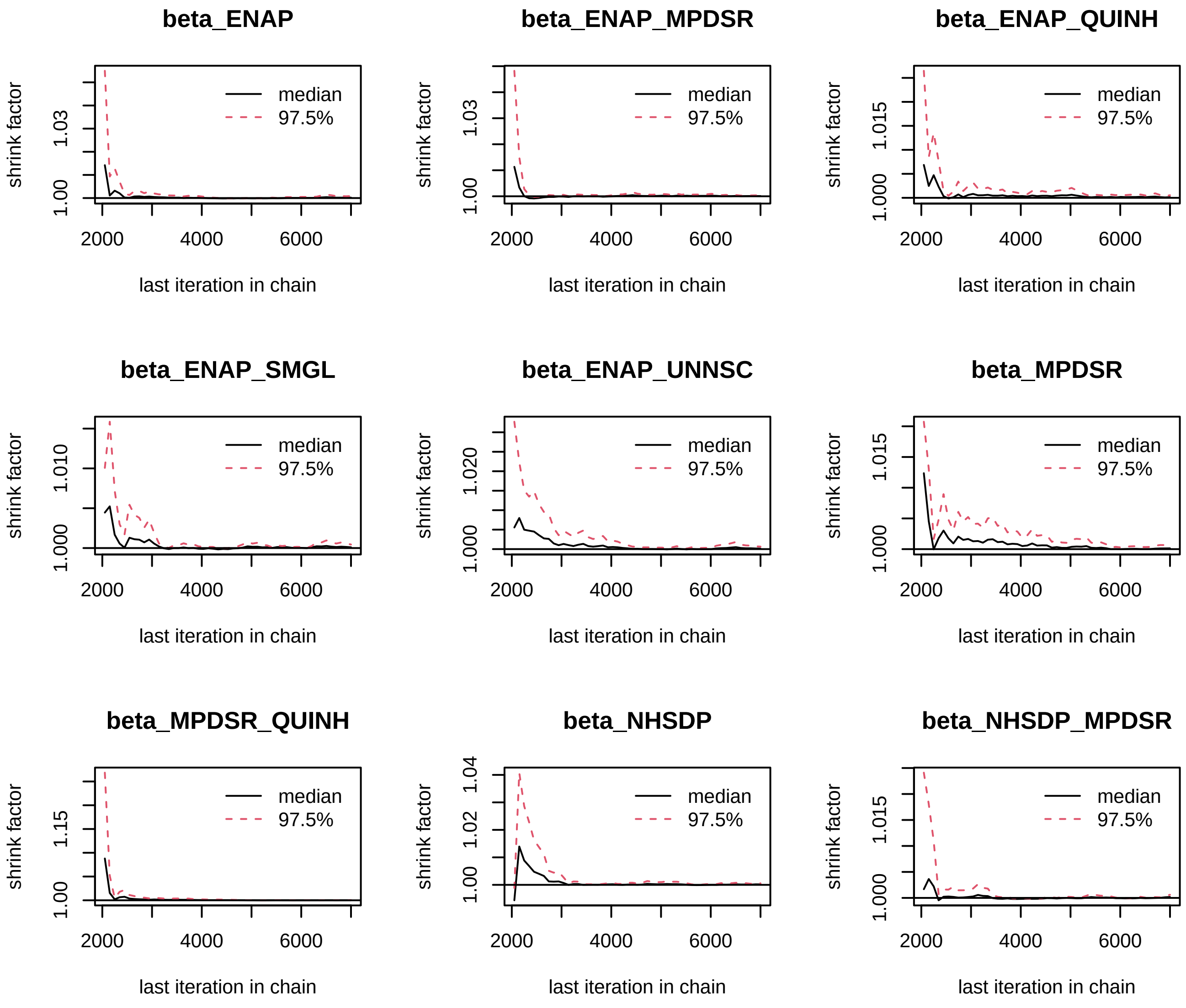
beta_ENAP
beta_ENAP_MPDSR
beta_ENAP_QUINH
beta_ENAP_SMGL
beta_ENAP_UNNSC
beta_MPDSR
beta_MPDSR_QUINH
beta_NHSDP
beta_NHSDP_MPDSR
median
97.5%
shrink factor
last iteration in chain
2000
4000
6000

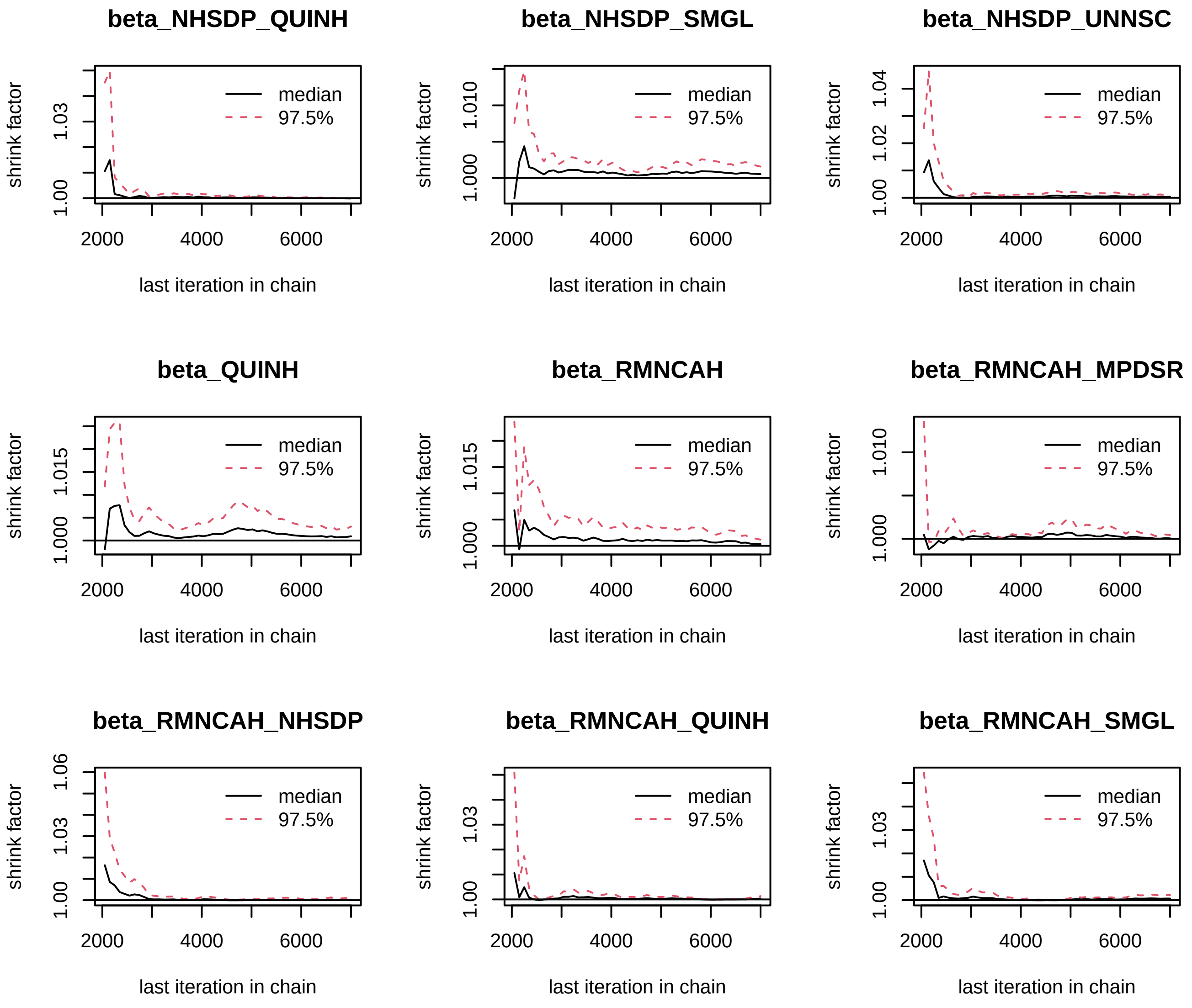
beta_NHSDP_QUINH
shrink factor
1.00
1.03
median
97.5%
2000
4000
6000
last iteration in chain
beta_NHSDP_SMGL
shrink factor
1.000
1.010
median
97.5%
2000
4000
6000
last iteration in chain
beta_NHSDP_UNNSC
shrink factor
1.00
1.02
1.04
median
97.5%
2000
4000
6000
last iteration in chain
beta_QUINH
shrink factor
1.000
1.015
median
97.5%
2000
4000
6000
last iteration in chain
beta_RMNCAH
shrink factor
1.000
1.015
median
97.5%
2000
4000
6000
last iteration in chain
beta_RMNCAH_MPDSR
shrink factor
1.000
1.010
median
97.5%
2000
4000
6000
last iteration in chain
beta_RMNCAH_NHSDP
shrink factor
1.00
1.03
1.06
median
97.5%
2000
4000
6000
last iteration in chain
beta_RMNCAH_QUINH
shrink factor
1.00
1.03
median
97.5%
2000
4000
6000
last iteration in chain
beta_RMNCAH_SMGL
shrink factor
1.00
1.03
median
97.5%
2000
4000
6000
last iteration in chain

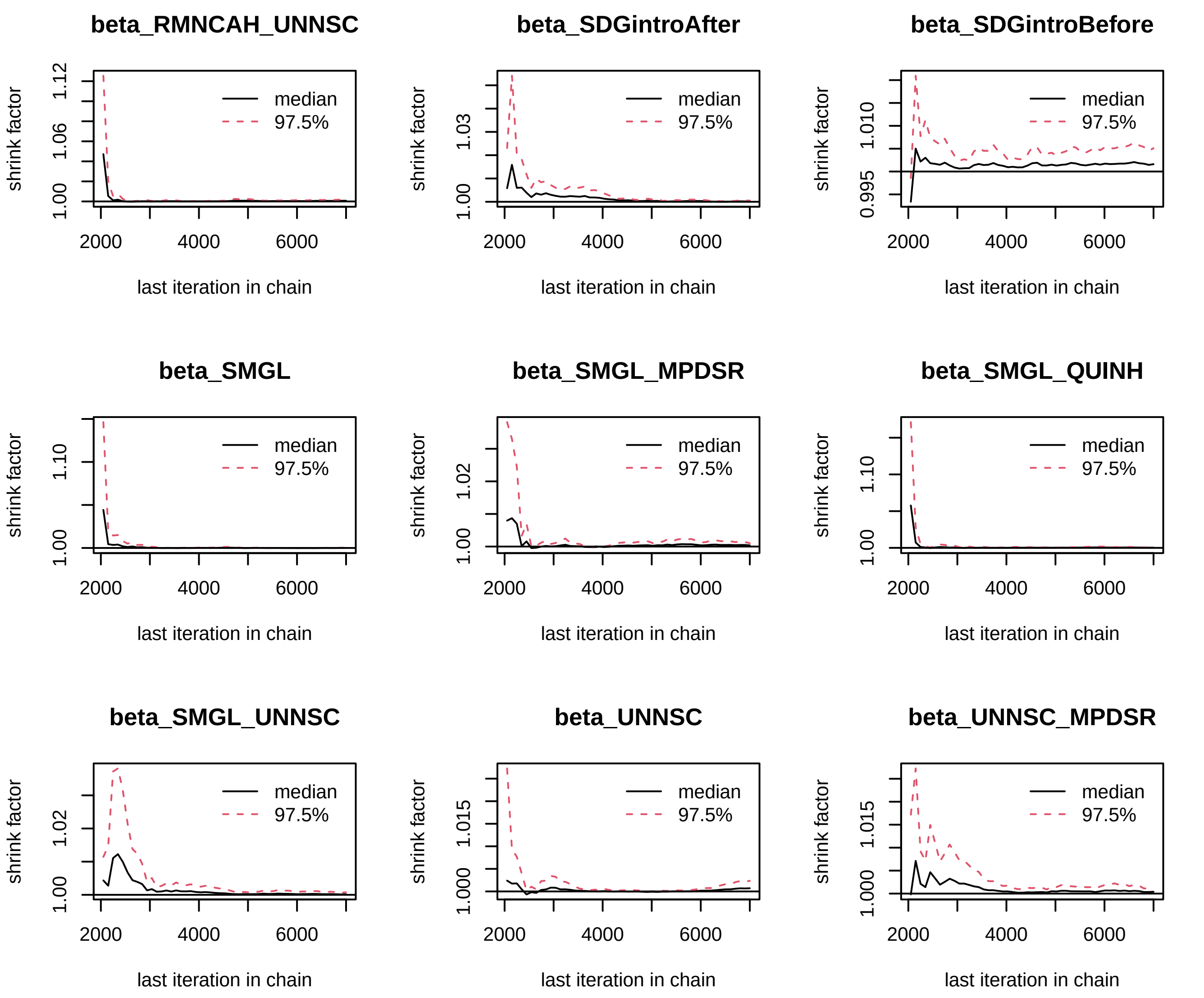
beta_RMNCAH_UNNSC
beta_SDGintroAfter
beta_SDGintroBefore
beta_SMGL
beta_SMGL_MPDSR
beta_SMGL_QUINH
beta_SMGL_UNNSC
beta_UNNSC
beta_UNNSC_MPDSR
shrink factor
last iteration in chain
median
97.5%
2000
4000
6000
1.00
1.06
1.12
1.03
0.995
1.010
1.10
1.02
1.000
1.015

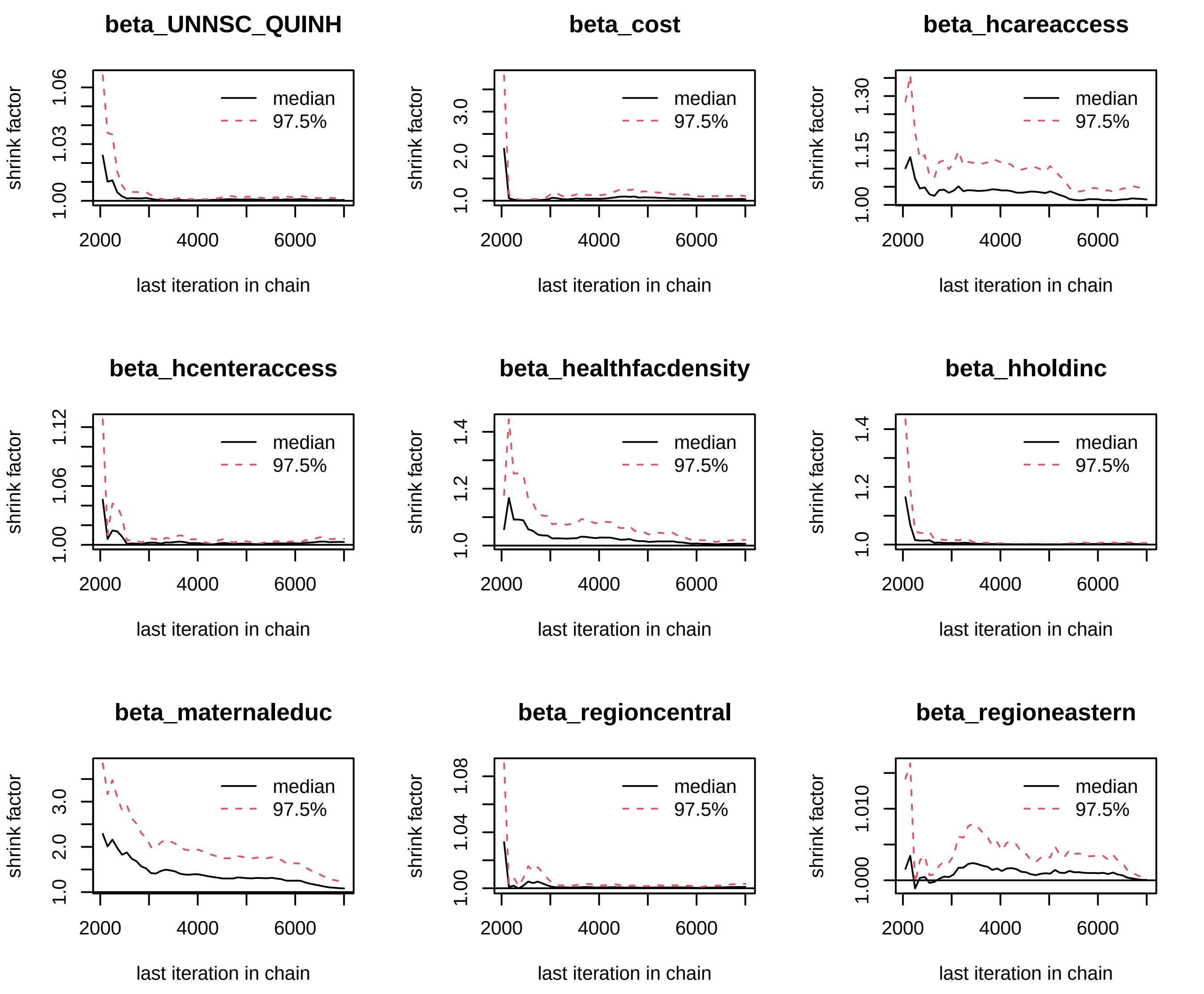
beta_UNNSC_QUINH
beta_cost
beta_hcareaccess
beta_hcenteraccess
beta_healthfacdensity
beta_hholdinc
beta_maternaleduc
beta_regioncentral
beta_regioneastern
shrink factor
last iteration in chain
median
97.5%
2000
4000
6000
1.00
1.03
1.06
1.0
2.0
3.0
1.15
1.30
1.12
1.2
1.4
1.04
1.08
1.000
1.010

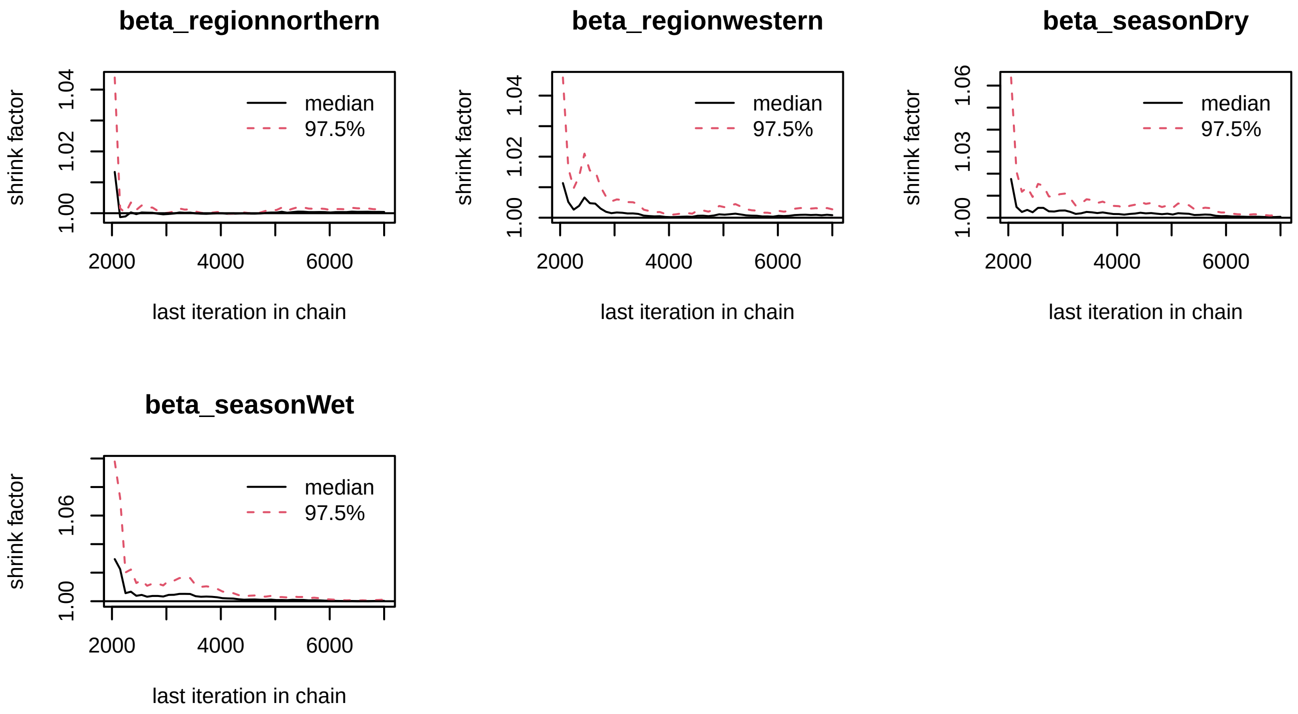
beta_regionnorthern
beta_regionwestern
beta_seasonDry
beta_seasonWet
shrink factor
median
97.5%
1.00
1.02
1.03
1.04
1.06
2000
4000
6000
last iteration in chain
